# Supplementary material for: A role for the S4-domain containing protein YlmH in ribosome-associated quality control in Bacillus subtilis
Source: Nucleic Acids Res. 2024 May 30;52(14):8483–99. doi: 10.1093/nar/gkae399 (PMC11317155; doi:10.1093/nar/gkae399)
Supplement: gkae399_Supplemental_Files [file gkae399_supplemental_files.zip › Takada_SI_HP2.pdf]

SUPPLEMENTARY ONLINE MATERIALS

for

A role for the S4-domain containing protein YlmH in ribosome-associated quality control in *Bacillus subtilis*

Hiraku Takada[1,2,3,#,*], Helge Paternoga[4,#,*], Keigo Fujiwara[1], Jose A. Nakamoto[3], Esther N. Park[5], Lyudmila Dimitrova-Paternoga[4], Bertrand Beckert[6], Merilin Saarma[7], Tanel Tenson[7], Allen R. Buskirk[5], Gemma C. Atkinson[3,8], Shinobu Chiba[1], Daniel N. Wilson[4], Vasili Hauryliuk[3,8,9]

[1] Faculty of Life Sciences, Kyoto Sangyo University and Institute for Protein Dynamics, Kamigamo, Motoyama, Kita-ku, Kyoto 603-8555, Japan

[2] Department of Biotechnology, Toyama Prefectural University,5180 Kurokawa, Imizu, Toyama 939-0398, Japan

[3] Department of Experimental Medical Science, Lund University, 221 00 Lund, Sweden

[4] Institute for Biochemistry and Molecular Biology, University of Hamburg, Martin-Luther-King-Platz 6, 20146 Hamburg, Germany

[5] Department of Molecular Biology and Genetics, Johns Hopkins University School of Medicine, Baltimore, MD, USA

[6] Dubochet Center for Imaging (DCI) at EPFL, EPFL SB IPHYS DCI, Lausanne, Switzerland

[7] University of Tartu, Institute of Technology, 50411 Tartu, Estonia

[8] Virus Centre, Lund University, Lund, Sweden

[9] Science for Life Laboratory, Lund, Sweden

[#] These authors contributed equally

*Correspondence to:
Hiraku Takada (hirakut58@pu-toyama.ac.jp),
Helge Paternoga (Helge.Paternoga@uni-hamburg.de

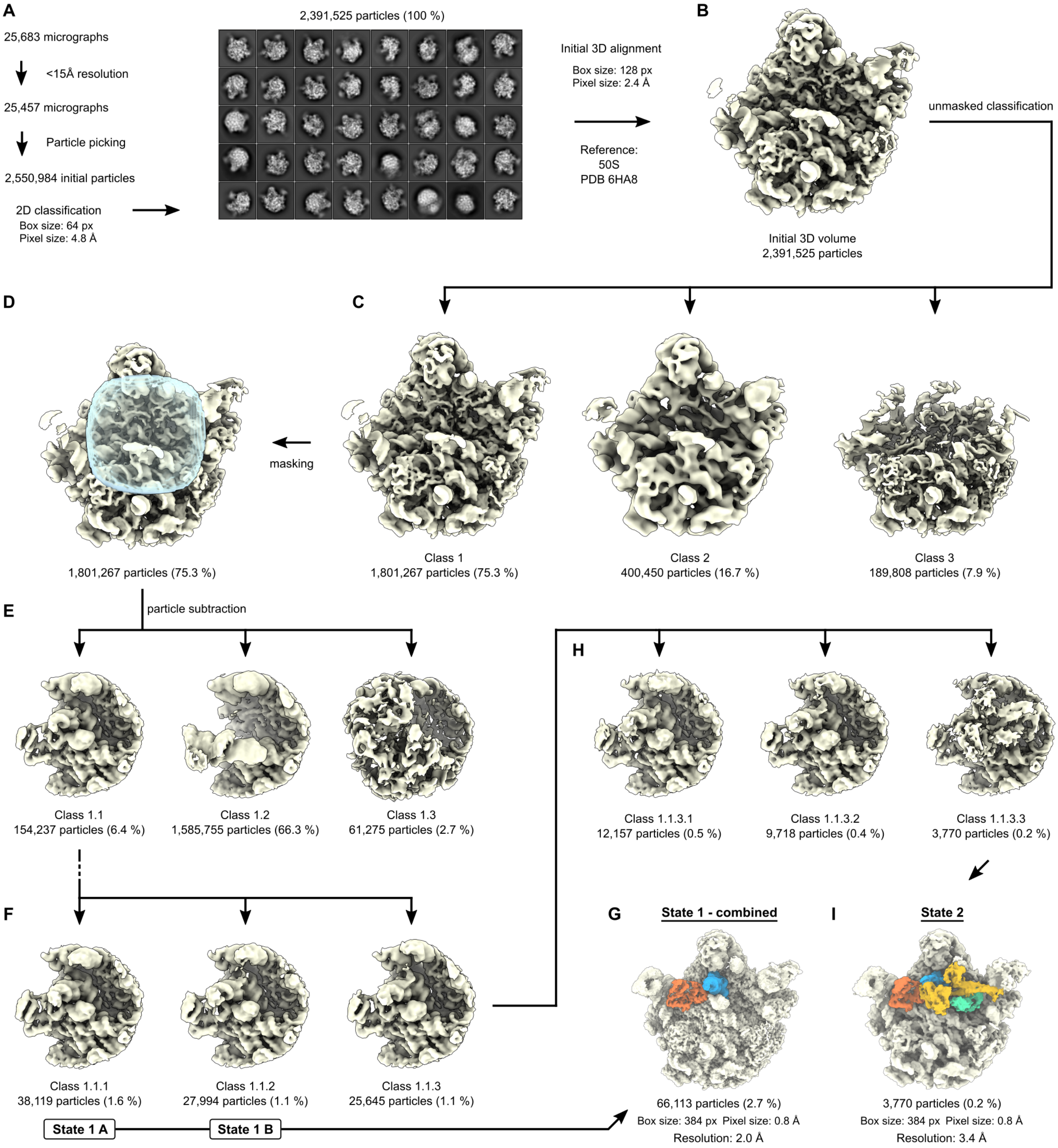

Supplementary Figure 1. Data processing of the YlmH-50S complexes. (**A**) 26,683 micrographs were collected and 25,457 remained after applying a resolution cut-off. 2,550,984 particles were selected from the micrographs and subjected to 2D classification. Of these 2,391,525 (termed 100%) were (**B**) 3D aligned and subjected to unmasked 3D classification, resulting in (**C**) three classes containing 50S or 50S-like particles. The major class 1 was (**D**) masked and subjected to 3D classification with particle subtraction, resulting in (**E**) three classes, only one of which (class 1) contained density for YlmH and P-tRNA. (**F**) Class 1 was further subjected to further 3D classification resulting in two well-defined states 1.1 and 1.2 (class 1 and 2) that were combined (66,113 particles, 2.7%), yielding (**G**) a final reconstruction of YlmH-P-tRNA-50S complex at 2.0 Å, which was termed State 1. (**H**) Class 3 was further subsorted into three classes, one of which (class 3 containing 3,770 particles, 0.2%) contained additional density that was further refined to

yield (**I**) a cryo-EM structure of the YlmH-RqcH-50S complex with A- and P-tRNAs at 3.4 Å, which was referred to as State 2.

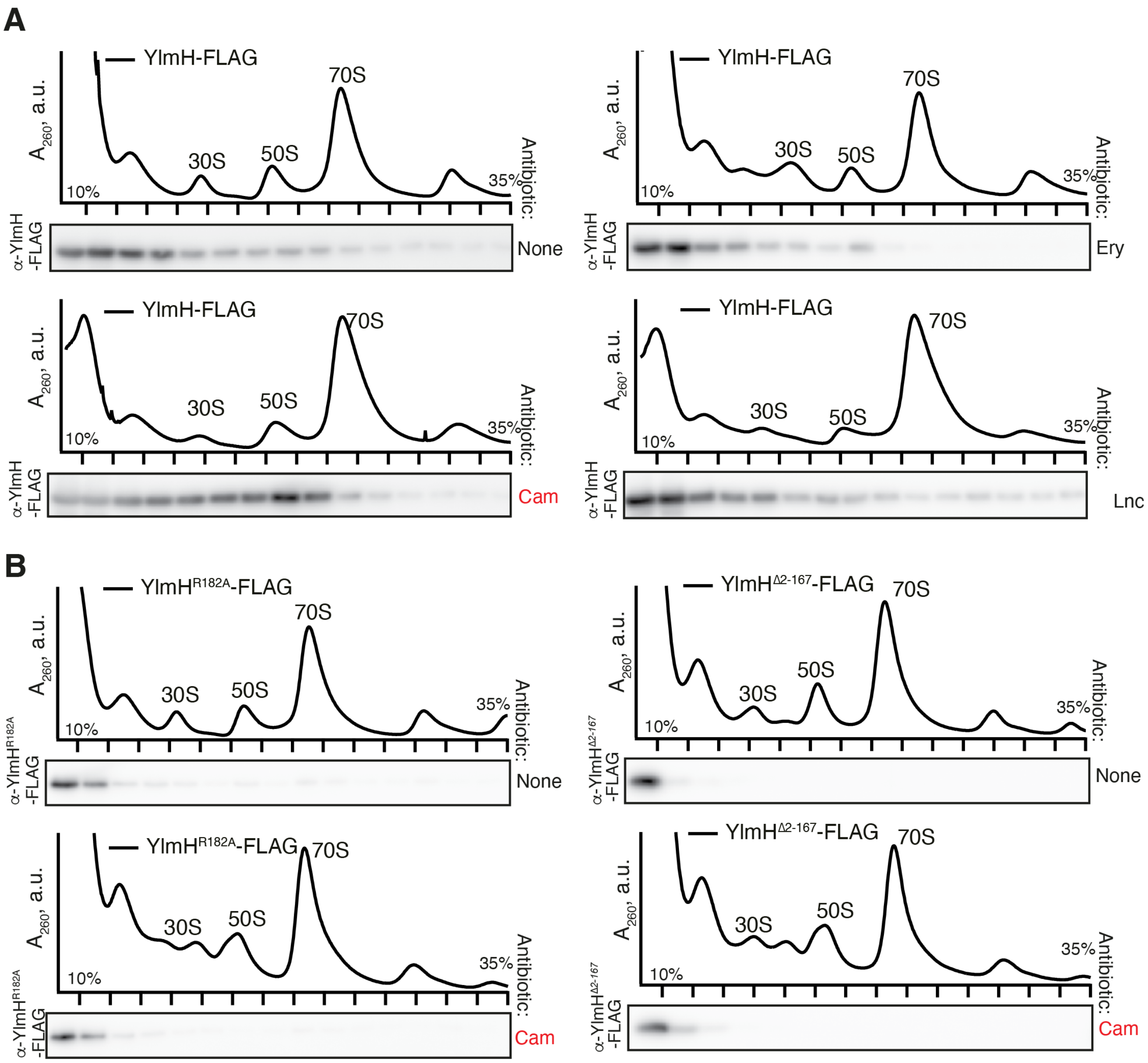

Supplementary Figure 2. Sucrose gradient analysis of YlmH variants with antibiotic treatment. (**A**) C-terminally FLAG-tagged YlmH (YlmH-FLAG; C-terminal FLAG tag connected via GS5 linker) was ectopically expressed in the Δ*ylmH* background under control of the $P_{hy\text{-}spank}$ promotor (BCHT931). YlmH expression was induced by addition of 30 µM IPTG. Following a 20-min antibiotic treatment with either chloramphenicol (Cam, 5 µg/mL), erythromycin (Ery, 1 µg/mL) or lincomycin (Lnc, 80 µg/mL), cellular lysates were resolved on sucrose gradients and fractions probed with anti-FLAG antibody. (**B**) C-terminally FLAG-tagged YlmH variants $YlmH^{R182A}$ and $YlmH^{\Delta 2\text{-}167}$ were ectopically expressed in the Δ*ylmH* background under control of the $P_{hy\text{-}spank}$ promotor. Following a 20-min antibiotic treatment with chloramphenicol (Cam, 5 µg/mL), cellular lysates were resolved on sucrose gradients and fractions probed with anti-FLAG antibody.

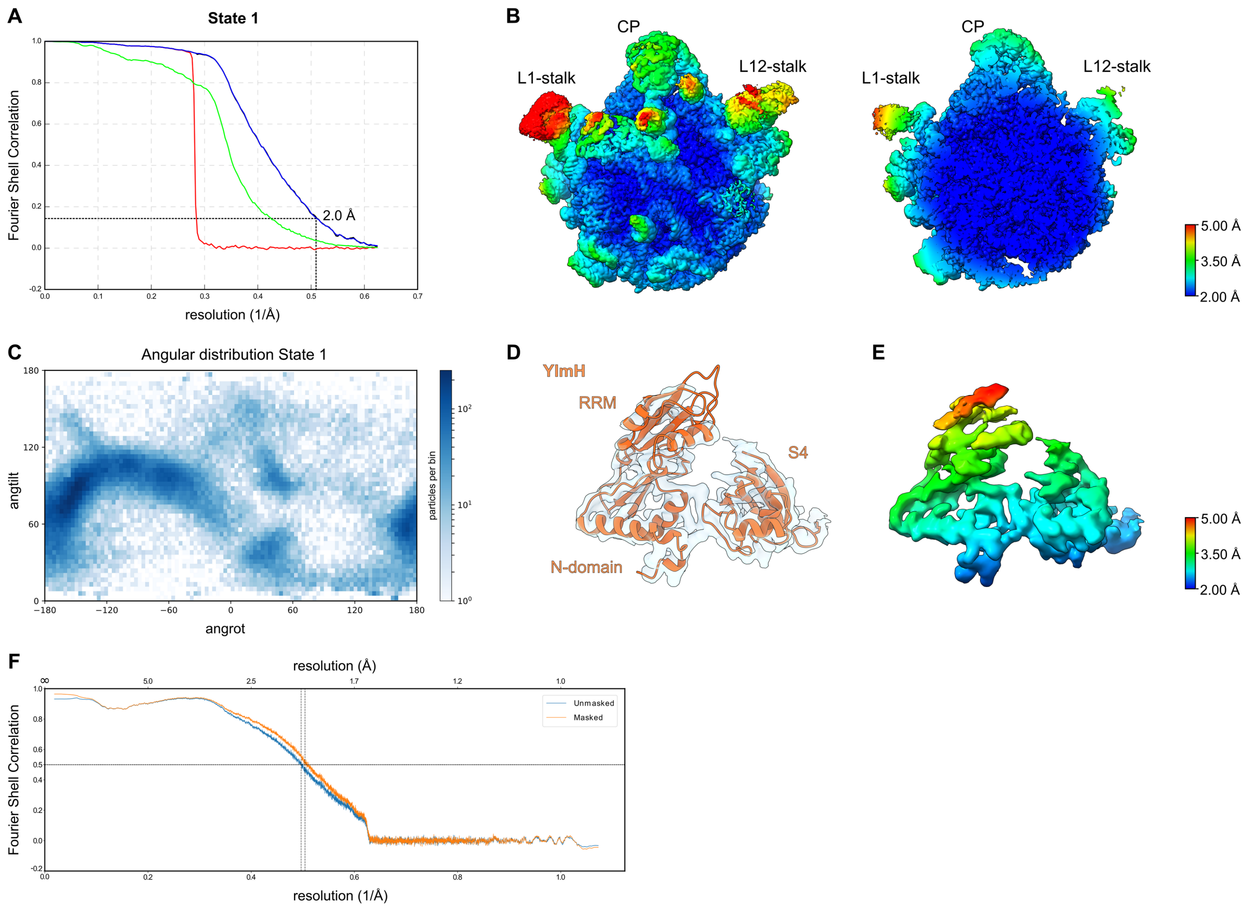

Supplementary Figure 3. Cryo-EM structure of the YlmH-50S complex (State 1). (**A**) Fourier Shell Correlation (FSC) curves for State 1 with the dashed line at 0.143 indicating an average resolution of 2.0 Å. The different curves include the masked map (green), unmasked map (blue), the phase-randomized masked map (red). (**B**) Cryo-EM map of State 1 colored according to local resolution with (left panel) interface overview and (right panel) transverse section revealing core of the 50S subunit. Landmarks for L1-stalk, L12-stalk and central protuberance (CP) are indicated. (**C**) Angular distribution of the particles that comprise State 1. (**D**) Isolated cryo-EM density (grey transparency) and molecular model (orange) for YlmH. (**E**) Isolated cryo-EM density as in (**D**) but coloured according to local resolution. (**F**) FSC map versus model with curves shown for unmasked (blue) and masked (orange) volumes.

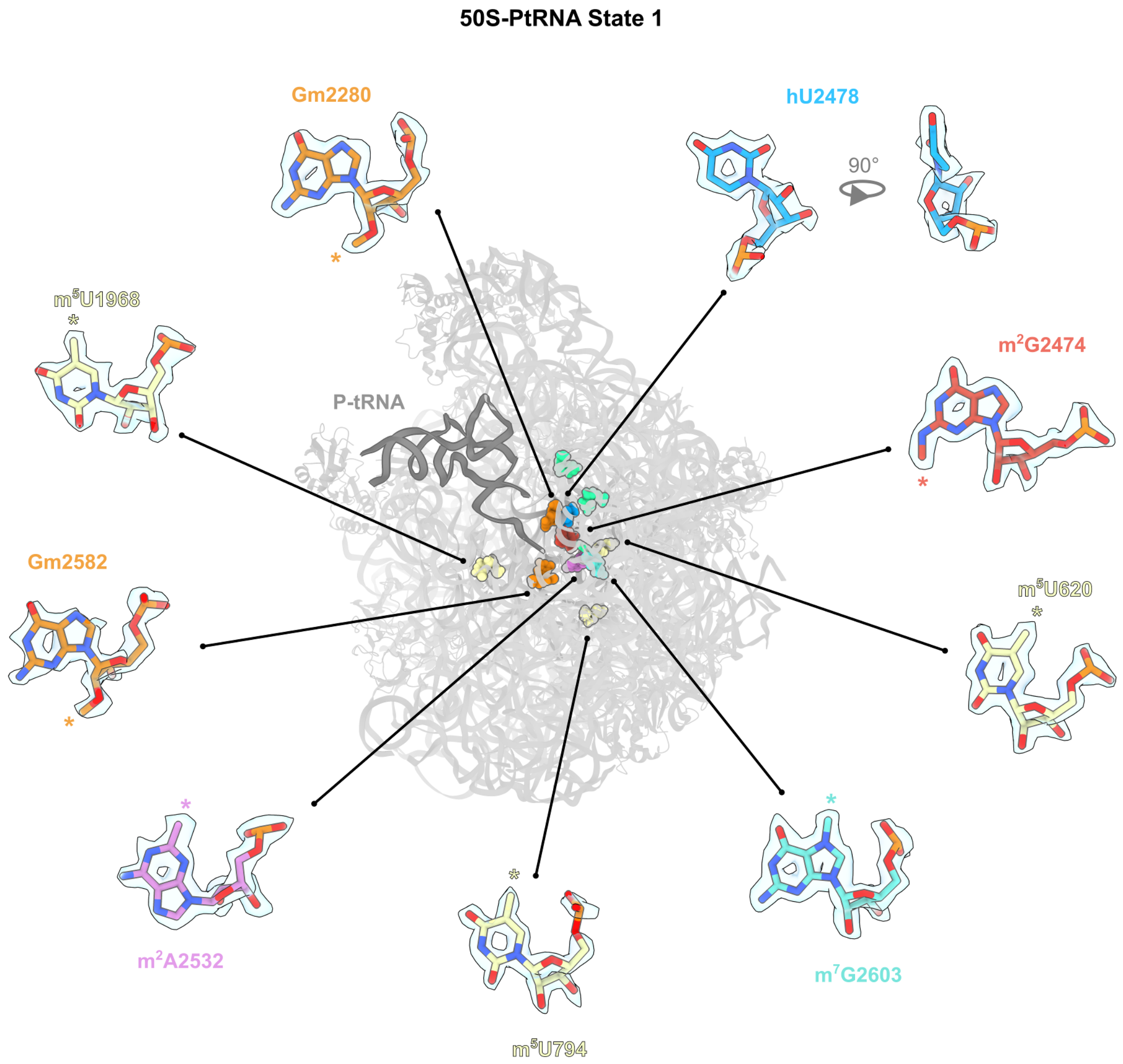

Supplementary Figure 4. Identification of rRNA modifications in the YlmH-50S complex (State 1). Sideview (from L1 side) of the 50S subunit (grey) with P-tRNA (dark grey) for reference, showing the position of 23S rRNA modifications identified in the cryo-EM of State 1. Enlargements show isolated cryo-EM density (grey transparency) with molecular models for each modification. Asterisks indicate the position of the modification.

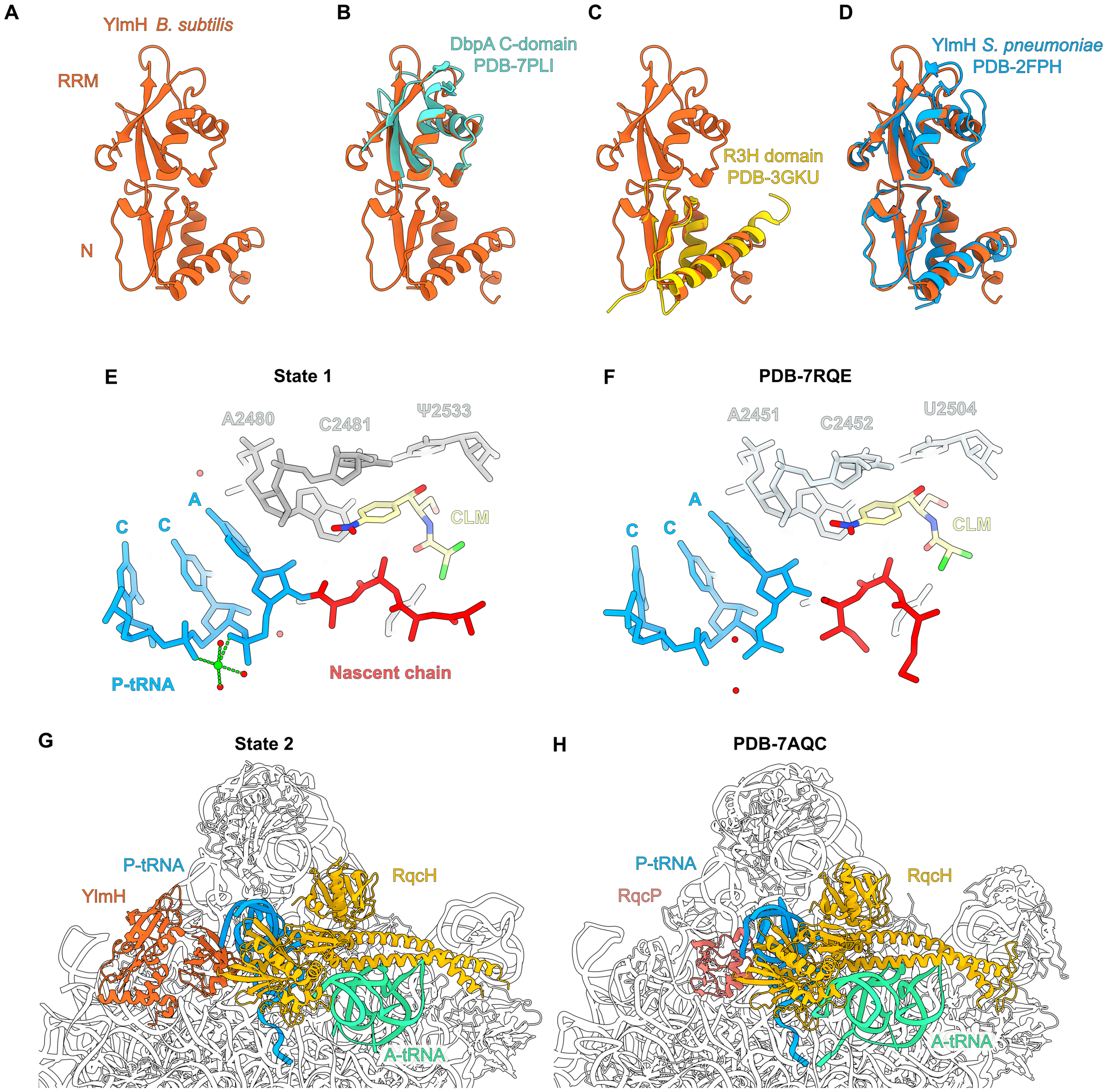

Supplementary Figure 5. Comparison of YlmH State 1 and 2 with previous structures. (**A-D**) Comparison of the RRM and N-domain of YlmH (orange) from State 1 with (**B**) the C-domain of DbpA (cyan, PDB ID 7PLI) (94), (**C**) the R3H domain of a probable RNA-binding protein from Clostridium symbiosum ATCC 14940 (PDB ID 3GKU), and (**D**) the crystal structure of the RRM and N-domain of YlmH from *Streptococcus pneumoniae* (PDB ID 2FPH). (**E-F**) Comparison of the CCA-end of the P-tRNA (blue), nascent chain (red), chloramphenicol (yellow) and selected 23S rRNA nucleotides (grey) from (**E**) YlmH-50S complex State 1 and (**F**) the crystal structure of the *Thermus thermophilus* 70S ribosome in complex with protein Y, A-site deacylated tRNA analog CACCA, P-site MAI-tripeptidyl-tRNA analog ACCA-IAM, and chloramphenicol (PDB ID 7RQE)(87). (**G-H**) Comparison of 50S subunit (white ribbons) in complex with YlmH (orange), RqcH (mustard), P-tRNA (blue) and A-tRNA (green) from (**G**) the YlmH-RqcH-50S complex with A- and P-tRNA (State 2), with (**H**) the structure of the bacterial RQC complex containing RqcP (orange), RqcH (mustard), P-tRNA (blue) and A-tRNA (green) (PDB ID 7AQC) (29).

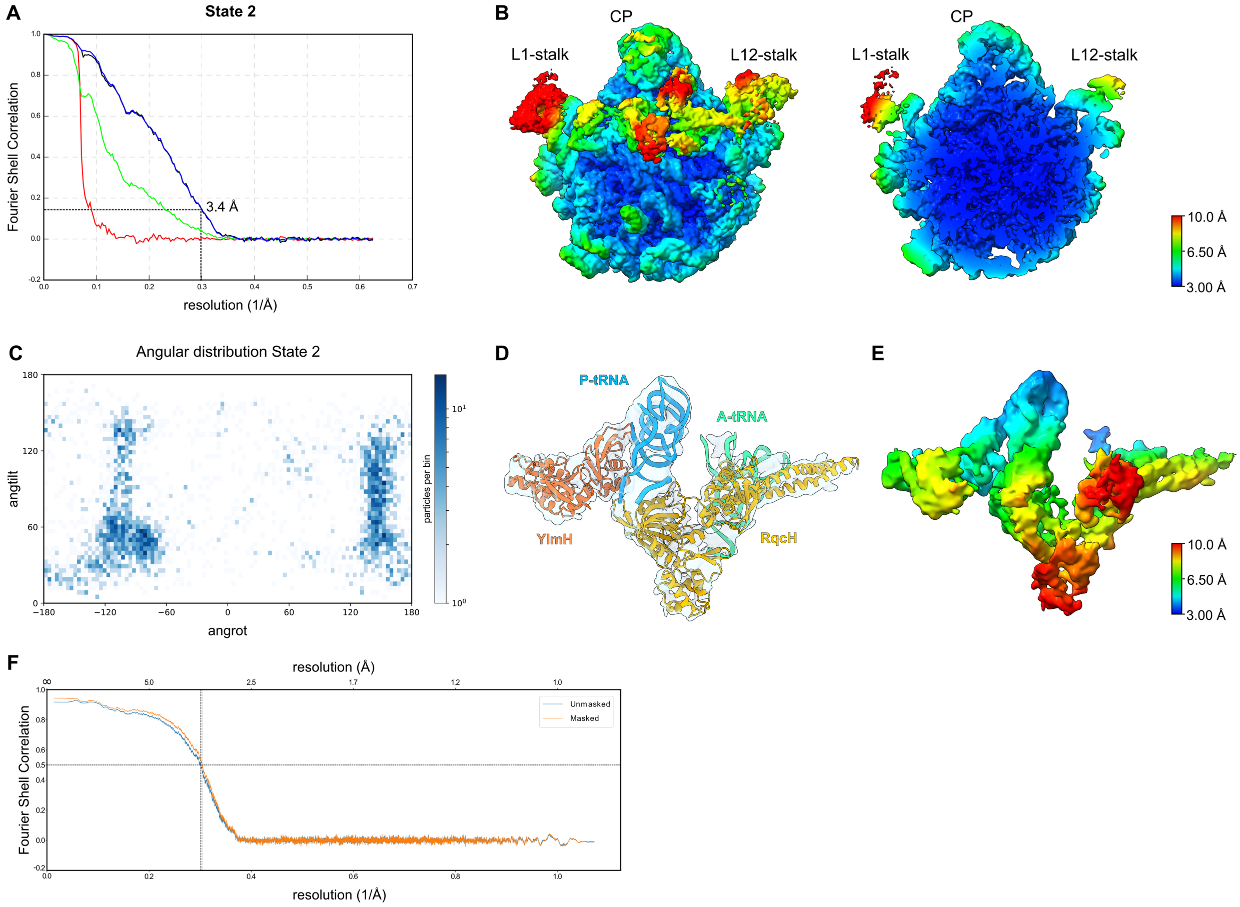

Supplementary Figure 6. Cryo-EM structure of the YlmH-RqH-50S complex (State 2). (**A**) Fourier Shell Correlation (FSC) curves for State 2 with the dashed line at 0.143 indicating an average resolution of 3.4 Å. The different curves include the masked map (green), unmasked map (blue), the phase-randomized masked map (red). (**B**) Cryo-EM map of State 2 colored according to local resolution with (left panel) interface overview and (right panel) transverse section revealing core of the 50S subunit. Landmarks for L1-stalk, L12-stalk and central protuberance (CP) are indicated. (**C**) Angular distribution of the particles that comprise State 2. (**D**) Isolated cryo-EM density (grey transparency) and molecular model for YlmH (orange), RqcH (mustard), P-tRNA (blue) and A-tRNA (green). (**E**) Isolated cryo-EM density as in (**D**) but coloured according to local resolution. (**F**) FSC map versus model with curves shown for unmasked (blue) and masked (orange) volumes.

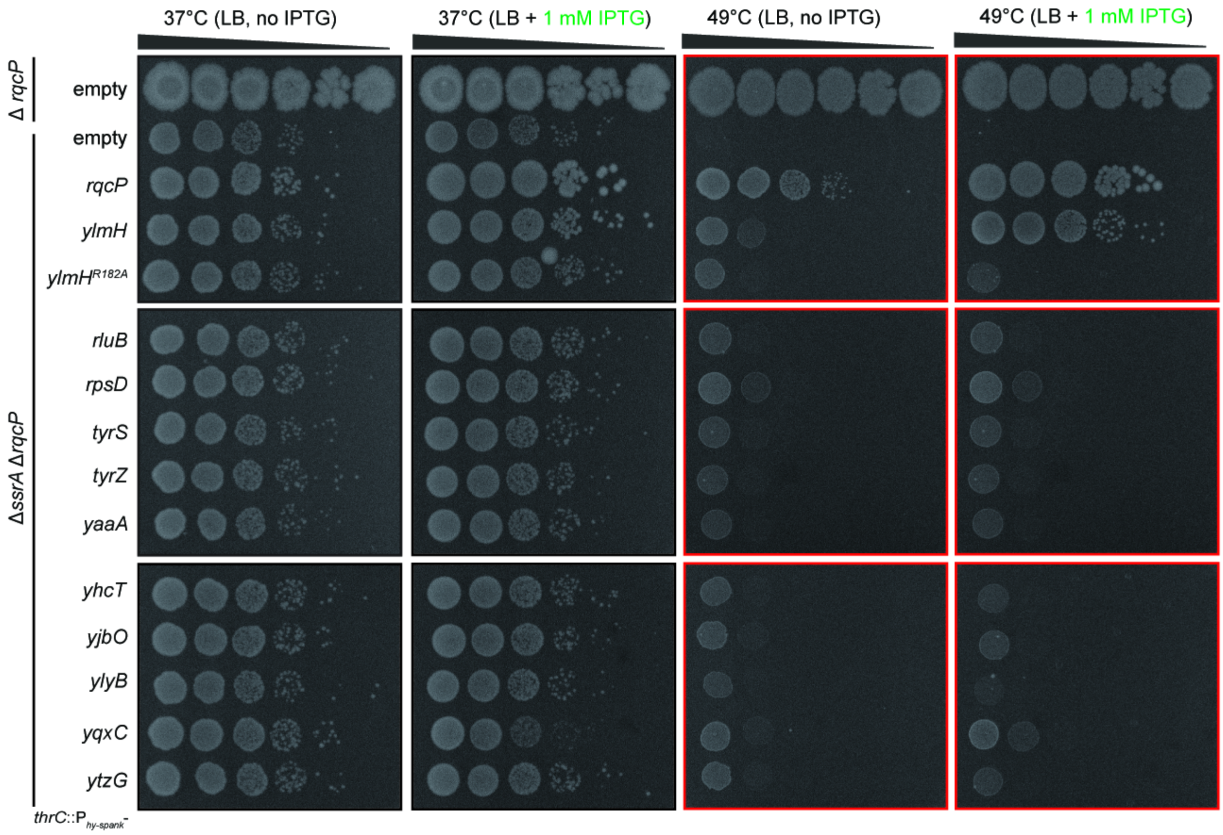

Supplementary Figure 7. Ectopic expression of YlmH but of not other tested *B. subtilis* S4 domain-encoding proteins suppresses the temperature sensitivity of the Δ*ssrA* Δ*rqcP* strain.

Expression of S4-domain-encoding *B. subtilis* proteins [negative control strain (BCHT1328), *rqcP* (BCHT1338), *ylmH* (BCHT1329), *ylmH*^{R182A} (BCHT1330), *rluB* (BCHT1332), *rpsD* (BCHT1339), *tyrS* (BCHT1340), *tyrZ* (BCHT1341), *yaaA* (BCHT1331), *yhcT* (BCHT1333), *yjbO* (BCHT1334), *ylyB* (BCHT1335), *yqxC* (BCHT1336) or *ytzG* (BCHT1337)] cloned under the control of $P_{hy\text{-}spank}$ promotor the in Δ*ssrA* Δ*rqcP* deletion background was induced by 1 mM IPTG. 10-fold serial dilutions were spotted onto LB agar plates with or without 1 mM IPTG and plates were scored after 18-hours incubation at either 37°C or 49°C.
